# Altered resting-state functional connectivity of the dorsal anterior cingulate cortex with intrinsic brain networks in male problematic smartphone users

**DOI:** 10.3389/fpsyt.2022.1008557

**Published:** 2022-10-03

**Authors:** Manjae Kwon, Young-Chul Jung, Deokjong Lee, Junghan Lee

**Affiliations:** ^1^Department of Psychiatry, Severance Hospital, Yonsei University College of Medicine, Seoul, South Korea; ^2^Institute of Behavioral Science in Medicine, Yonsei University College of Medicine, Seoul, South Korea; ^3^Institute for Innovation in Digital Healthcare, Yonsei University, Seoul, South Korea; ^4^Department of Psychiatry, Yongin Severance Hospital, Yonsei University College of Medicine, Seoul, South Korea

**Keywords:** attention network, default mode network, problematic smartphone use, functional MRI, dorsal anterior cingulate cortex

## Abstract

The excessive use of smartphones is associated with various medical complications and mental health problems. However, existing research findings on neurobiological mechanisms behind problematic smartphone use are limited. In this study, we investigated functional connectivity in problematic smartphone users, focusing on the default mode network (DMN) and attentional networks. We hypothesized that problematic smartphone users would have alterations in functional connectivity between the DMN and attentional networks and that such alterations would correlate with the severity of problematic smartphone use. This study included 30 problematic smartphone users and 35 non-problematic smartphone users. We carried out group independent component analysis (group ICA) to decompose resting-state functional magnetic resonance imaging (fMRI) data into distinct networks. We examined functional connectivity using seed-to-seed analysis and identified the nodes of networks in group ICA, which we used as region of interest. We identified greater functional connectivity of the dorsal anterior cingulate cortex (dACC) with the ventral attention network (VAN) and with the DMN in problematic smartphone users. In seed-to-seed analysis, problematic smartphone users showed atypical dACC-VAN functional connectivity which correlated with the smartphone addiction proneness scale total scores. Our resting-state fMRI study found greater functional connectivity between the dACC and attentional networks in problematic smartphone users. Our findings suggest that increased bottom-up and interoceptive attentional processing might play an important role in problematic smartphone use.

## Introduction

Smartphone use has increased greatly over the last decade, and has become an integral part of our daily lives. Whilst a smartphone has brought about great convenience, its excessive use has also brought many adverse health effects on its user (1–3). Problematic smartphone use has been associated with depression and anxiety (4). It has also been associated with psychological distress mediated by emotional dysregulation (5), with higher impulsivity and higher alcohol use disorder symptoms (6).

Recent studies have demonstrated similarities between excessive smartphone usage and behavioral addiction (7, 8). The Diagnostic and Statistical Manual of Mental Disorders-5 (DSM-5) diagnostic criteria for behavioral addiction are similar to that of substance use disorder and include symptoms of withdrawal and tolerance, continued use despite negative consequences and loss of control over the activity (9). Researchers observed that problematic smartphone use share common symptomatology with other behavioral addiction, leading to have a negative impact on functioning in occupational, social, and daily life (10). As Kardefelt-Winther et al. proposed, two important components in behavioral addictions are: (a) significant functional impairment or distress as a direct consequence of the behavior and (b) persistence over time. Accordingly, functional impairment from excessive smartphone use has been demonstrated through academic problems, interpersonal problems, impairment of cognitive function leading to reduced inhibitory control, impaired attention or in reduced numerical processing capacity (11–13).

Researchers have attempted to elucidate neurobiological mechanisms behind behavioral addictions. Between behavioral addiction and substance addiction, shared alteration in neurocircuitry, especially in the frontal and striatal regions has been demonstrated in neuroimaging studies (14–16). Mesolimbic pathway has been shown to be associated with both substance addiction and behavioral addiction (17, 18). Attentional control, which is a key factor in exerting executive function, has been associated with the severity of gambling in problem gamblers (19). Attentional control involves two systems, namely top-down factors and bottom-up factors. The dorsal attention network (DAN) is in charge of top-down attentional control and takes into account knowledge, expectation and current goals (20). The DAN includes the intraparietal sulcus and frontal eye field (20). The ventral attention network (VAN) consists of the ventral frontal cortex and temporoparietal junction and is in charge of bottom-up attentional control (21). Hypoconnectivity within the VAN has also been shown in subjects with internet addiction (22). Moreover, the default mode network (DMN) is a large-scale network that is activated during resting state but deactivated when performing task. The DMN comprises of the medial prefrontal cortex, posterior cingulate cortex and precuneus in the midline of the brain and the bilateral inferior parietal lobule (23). Altered functional connectivity in the DMN has been demonstrated in internet addiction and internet gaming disorder (24, 25).

There are a small number of studies that examine the neurobiological mechanisms behind problematic smartphone use. To consider a number of existing studies – structural magnetic resonance imaging (MRI) studies have demonstrated that problematic smartphone users exhibit smaller gray matter volume in the right orbitofrontal cortex (26). Hovarth et al. in their amplitude of low frequency fluctuation (ALFF) study, demonstrated that intrinsic activity in the right anterior cingulate cortex was negatively correlated with the smartphone addiction inventory (SPAI) (27). Subjects with smartphone addiction also showed significantly smaller gray matter volume in the insula compared to controls (27). Chun et al. in resting-state functional magnetic resonance imaging (fMRI) study using region of interest (ROI) to ROI analysis, observed that adolescents with excessive smartphone use showed lower functional connectivity between the orbitofrontal cortex and nucleus accumbens, and between the orbitofrontal cortex and middle cingulate cortex (28).

The majority of the existing functional connectivity studies on problematic smartphone users are based on *a priori* selection of ROIs, which may introduce potential bias and limit studies from having a global view of the brain connectivity (29). While it is still an open question to decide which method is superior, previous studies demonstrated that when researchers define ROI using brain atlas, the selected ROIs may not correspond to the real functional boundaries and that using data-driven independent component analysis (ICA) would be more appropriate (30).

In this study, we investigated alterations in functional connectivity in male problematic smartphone users by using group independent component analysis (group ICA), a data-driven technique, which can discover hidden factors from a set of measurements without *a priori* selection of ROIs (31), focusing on the DMN and attentional networks. We defined ROIs from group ICA results, as it may better correspond to the real functional boundaries for the data collected in our study (30). We hypothesized that problematic smartphone users would have alterations in functional connectivity in the DMN and in attention networks, and that such alterations will correlate with the severity of problematic smartphone use.

## Materials and methods

### Participants

A total of 65 male smartphone users participated in the study. As previous studies demonstrated gender differences in the neurobiology underlying addictions (32–36), and as brain imaging studies have shown structural and functional differences between male and female participants with addictive disorders (37), we have only gathered male participants, in an effort to collect a more homogenous sample, especially considering the small number of participants in our study.

We recruited participants from the local community through announcements, flyers or word of mouth. We administered the Structured Clinical Interview for DSM-5 to evaluate major psychiatric illness (38). The Korean version of the Wechsler Adult Intelligence Scale IV was administered on all participants to assess intelligence quotient (IQ) (39). Exclusion criteria were: participants with major psychiatric disorder, intellectual disability, neurological illness or contraindication to MRI. Participants included in the study had no history of receiving psychiatric treatment or taking psychiatric medication. Participants were divided into two groups: problematic smartphone users and non-problematic smartphone users.

### Psychometric measures

Participants were assessed for excessive smartphone use using the Korean Smartphone Addiction Proneness Scale (SAPS) (40). The SAPS, developed by the Korean National Information Society Agency, is used to evaluate excessive smartphone use (it shows high reliability with Cronbach’s alpha of 0.880) (40). The scale consists of four subdomains: disturbance of adaptive functions, virtual life orientation, withdrawal and tolerance. The scale also consists of 15 items, rated by the 4-point Likert scale (1 = strongly disagree; 2 = disagree; 3 = agree; 4 = strongly agree) ranging from 15 to 60. Participants with a total SAPS score of ≥44 or disturbance of adaptive functions, withdrawal and tolerance subscale scores of ≥15, ≥13, and ≥13, respectively were classified as high-risk smartphone users. At-risk smartphone users were defined as participants with the SAPS score of 40–43 or disturbance of adaptive functions subscale score of ≥14. In our study, we classified both high-risk and at-risk smartphone users as problematic smartphone users, following what was proposed during the SAPS development (41), which is also in line with the classification criteria used in previous studies (26, 28). To examine internet addiction status, the Internet Addiction Test (IAT) was administered (42). To assess impulsivity, the Barratt Impulsiveness Scale (BIS) was administered (43). All subjects were assessed with the Beck Depression Inventory (BDI) (44), Beck Anxiety Inventory (BAI) (45), Alcohol Use Disorder Identification Test (AUDIT) (46), Pittsburgh Sleep Quality Index (PSQI) (47), Conners’ Adult ADHD Rating Scale-Short Version (CAARS) (48) and Wender Utah Rating Score-Short Version (WURS) (49).

### Image acquisition and pre-processing

We used a 3T MRI scanner (Magnetom; Siemens, Munich, Germany) equipped with an eight-channel head coil to acquire brain MRI data. To acquire structured MRI data, we used T1-weighed spoiled gradient echo sequence (echo time = 2.19 ms, repetition time = 1,780 ms, flip angle = 9°, field of view = 256 mm × 256 mm, matrix = 256 × 256, transverse slice thickness = 1 mm). We obtained fMRI data through T2-weighed gradient echo planar pulse sequence (echo time = 30 ms, repetition time = 2,500 ms, flip angle = 90°, field of view = 240 mm × 240 mm, matrix = 64 × 64, slice thickness = 3.5 mm). T2-weighed sequence was used for this fMRI experiment because the transverse relaxation time (T2) is dependent on blood oxygenation level (50, 51). We instructed participants to fixate on a white cross on a black background for 6 min, without any motor, cognitive or lingual activities.

We used CONN-fMRI functional connectivity toolbox, version 20.b^1^ for preprocessing functional connectivity analysis. We applied CONN preprocessing pipeline. Using the Statistical Parametric Mapping (SPM12; Wellcome Trust Centre for Neuroimaging)^2^ realign and unwarp procedure (52), we realigned the functional data. We also applied unwarping and slice-timing correction.

We then ran Artifact Detection Tool (ART)-based automatic outlier detection. If functional volume signal intensity was outside five standard deviations from the mean, or if there was any evidence that they were displaced for more than 0.9 mm compared to the preceding volume, they were considered outliers. If we needed to censor more than 15% of frames for scrubbing, we excluded the relevant participant. All participants were included in the functional connectivity analysis as they did not meet the exclusion criteria. SPM12 unified segmentation and normalization procedure (53) was used to normalize functional and anatomical data into standard Montreal Neurological Institute (MNI) space and to segment them into gray matter, white matter and cerebrospinal fluid. In this process, we iteratively performed tissue classification, estimating the posterior tissue probability maps (TPMs) from the intensity values of the reference image and registration, estimating the non-linear spatial transformation best approximating posterior and prior TPMs until convergence. We re-sliced images to a 2-mm isotropic resolution. Images were smoothed with an 8-mm full-width at half maximum kernel.

### Functional connectivity analysis

We obtained functional connectivity maps of seed-to-seed analysis using the CONN-fMRI functional connectivity toolbox version 20.b. For functional connectivity analysis, seed regions were selected from results of group ICA. The seed regions were selected from the largest clusters of each components of interest with peak *z*-values. ROIs were defined as a 6-mm radium sphere centered on coordinates acquired from spatial maps of targeted functional network.

### Group independent component analysis

We carried out group ICA to investigate spatially independent network using group ICA of fMRI toolbox (GIFT ver 4.0c).^3^ Two stages of principal component analysis were performed to reduce the preprocessed data. Dimensionality estimation to determine the number of independent components from fMRI data was performed using the modified minimal description length criteria (54). This method has been widely used in ICA studies using fMRI data (55–57). We repeated ICA analysis 20 times and obtained 38 independent functional spatial maps for every participant.

We selected ICA components through stepwise estimation. Firstly, we used probabilistic maps of white matter, cerebrospinal fluid within standardized brain space in MNI template in SPM12 and correlated it with each component. Spatial correlation greater than *r*^2^ = 0.025 in both white matter and cerebrospinal fluid were considered artifacts and were therefore removed from the analysis (Supplementary Figures 4, 5). Secondly, we selected candidates of large-scale intrinsic networks, and remaining components were correlated with network atlas of the DMN (58). To analyze within and between group differences, we performed one-sample *t*-tests and two-sample *t*-tests using SPM12 with “SPM stat” function in the GIFT toolbox. We converted spatial maps of selected components to *Z*-values to assess correlations among components.

### Statistical analysis

We performed independent *t*-test to compare demographic and clinical variables between problematic smartphone users and non-problematic smartphone users. We examined the relationship between clinical assessments and functional connectivity using the Pearson correlation analysis. The Statistical Package for the Social Sciences (SPSS), version 26.0 (SPSS Inc., Chicago, IL, USA) was used for statistical analysis with *p*-value < 0.05 (two-tailed).

### Ethics

The Institutional Review Board at Severance Hospital, Yonsei University approved all protocols for this study. Written informed consent was obtained from all participants prior to participation.

## Results

### Demographic and clinical assessment

There were no differences between the two groups in age and in IQ. The SAPS score was significantly higher in problematic smartphone users. The total score in BIS did not show any significant difference between problematic smartphone users and non-problematic smartphone users, while non-planning impulsiveness subscale was higher in problematic smartphone users. The total score in CAARS did not differ between problematic smartphone users and non-problematic smartphone users, while hyperactivity, restlessness subscale score was higher in problematic smartphone users. The PSQI score was significantly higher in problematic smartphone users (Table 1).

**TABLE 1:** Demographic and clinical characteristics of participants.

	Problematic smartphone users	Non-problematic smartphone users	T	*p*
**Age (years)**	30.3(2.1)	35(2.0)	0.475	0.636
SAPS*	48.9(5.4)	32.5(6.3)	11.2	<0.001
**IQ**	103.6(10.5)	101.1(12.5)	0.860	0.393
**BDI**	13.4(8.4)	11.7(9.2)	0.791	0.432
**BAI**	12.9(10.0)	9.4(9.4)	1.467	0.147
**BIS**	55.9(9.2)	51.5(8.8)	1.955	0.055
Cognitive impulsiveness	22.9(4.2)	21.4(4.3)	1.355	0.180
Motor impulsiveness	17.1(4.5)	15.7(3.4)	1.408	0.164
Non-planning impulsiveness*	16.0(2.8)0	14.4(2.4)	2.390	0.020
**AUDIT**	12.2(10.0)	9.2(5.1)	1.456	0.153
**CAARS**	25.1(12.8)	20.2(11.4)	1.637	0.107
Inattention/memory problems	6.2(2.9)	5.1(3.6)	1.408	0.165
Hyperactivity/restlessness*	6.6(3.6)	5.0(2.8)	2.008	0.049
Impulsivity/emotional lability	4.8(4.3)	3.7(3.7)	1.107	0.273
Problems with self-concept	7.6(4.0)	6.5(4.0)	1.054	0.296
**WURS**	35.9(25.5)	35.5(19.6)	0.073	0.942
PSQI*	9.1(2.1)	7.5(2.9)	2.483	0.016
Sleep quality	1.7(0.7)	1.4(0.7)	1.727	0.089
Sleep latency*	2.2(0.9)	1.6(1.0)	2.567	0.013
Sleep duration	1.2(1.0)	1.0(1.0)	0.685	0.496
Habitual sleep efficiency	0.6(0.9)	0.4(0.6)	1.414	0.164
Sleep disturbances	1.2(0.6)	1.1(0.5)	0.687	0.495
Use of sleep medication	0.1(0.3)	0.1(0.6)	−0.379	0.706
Daytime dysfunction	2.0(0.9)	1.8(0.7)	1.034	0.305

Values are expressed as mean (SD). Intelligence Quotient (IQ) was assessed with Wechsler Adult Intelligence scale, SAPS, Smartphone Addiction Proneness Scale; BDI, Beck Depression Inventory; BAI, Beck Anxiety Inventory; BIS, Barratt Impulsiveness Scale; AUDIT, Alcohol Use Disorder Identification Test; PSQI, Pittsburgh Sleep Quality Index; CAARS, Conners’ Adult ADHD Rating Scale-Short Version; WURS, Wender Utah Rating Score-Short Version. *p-value < 0.05.

### Group independent component analysis

Among the 38 independent components, 7 components passed our selection criteria. The selected components showed a low correlation with white matter and cerebrospinal fluid, and were highly correlated with gray matter and pre-existing templates (58, 59) (Supplementary Figures 1, 2).

**FIGURE 1:**
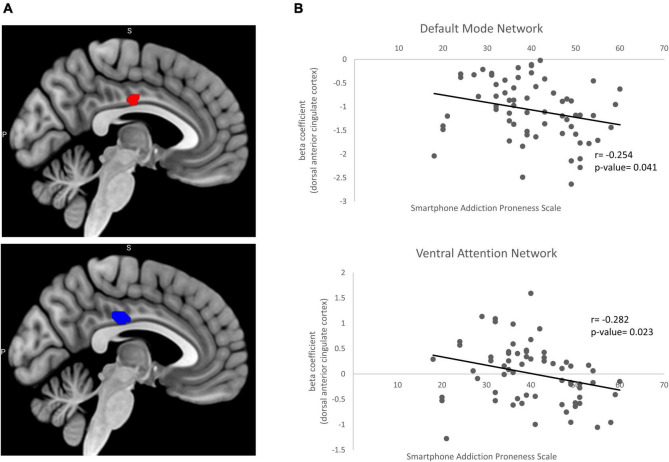
Beta coefficient from two sample *t*-test and correlation with clinical variable. **(A)** Significant increases in the resting state functional connectivity of the default mode network (red) and ventral attention network (blue) in problematic smartphone users. Problematic smartphone users displayed increased resting state functional connectivity in dorsal anterior cingulate cortex in both default mode network and ventral attention network. **(B)** Pearson correlation analysis for correlation of beta coefficient in dorsal anterior cingulate cortex and smartphone addiction proneness scale. In default mode network, beta coefficient in dorsal anterior cingulate cortex showed negative correlation (Pearsons’s *r* = –0.254, *p*-value = 0.041). In ventral attention network dorsal anterior cingulate cortex also showed negative correlation with smartphone addiction proneness scale (Pearson’s *r* = –0.282, *p*-value = 0.023).

The DMN (IC 21) consists of posterior cingulate cortex, angular gyrus and medial prefrontal cortex. The VAN (IC 37) consists of inferior frontal gyrus and temporoparietal junction. The right DAN (IC 32) and the left DAN (IC 20) consist of intraparietal sulcus and frontal eye field. In two-sample *t*-test of the DMN and VAN, the left dorsal anterior cingulate cortex (dACC) was hyperactivated in problematic smartphone user group compared to non-problematic smartphone user group. We attempted to find out whether the activation of the dACC was associated with proneness to smartphone addiction by examining the correlation between the SAPS score and beta weights of the dACC ROI from the DMN and VAN. Beta weights of the left dACC ROI selected from the DMN showed a negative correlation with the SAPS score (Pearson’s r: −0.254, *p*-value = 0.041) (Figure 1A). Beta weights of the left dACC ROI from the VAN also showed a negative correlation with the SAPS score (Pearson’s *r* = −0.282, *p*-value = 0.023) (Figure 1B).

### Functional connectivity analysis

**FIGURE 2:**
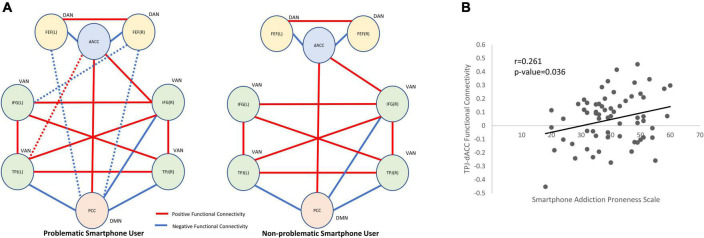
Between-network connectivity and functional connectivity correlation with clinical variables. **(A)** Seed-to-seed within-group analysis (red line: positive functional connectivity; blue line: negative functional connectivity). The statistical inferences were thresholded using a *p*-value < 0.05. In problematic smartphone users, functional connectivity of dACC to left TPJ was identified which were not observed in non-problematic smartphone users. Functional connectivity of PCC to FEF was found in problematic smartphone user group, which were not observed in non-problematic smartphone user group. **(B)** Pearson correlation analysis for clinical correlation of functional connectivity. We identified positive correlation between TPJ-dACC functional connectivity and smartphone addiction proneness scale (Pearson’s *r* = 0.261, *p*-value = 0.036). DAN, dorsal attention network; VAN, ventral attention network; DMN, default mode network; dACC, dorsal anterior cingulate cortex; IFG, inferior frontal gyrus; TPJ, temporoparietal junction, PCC, posterior cingulate cortex; FEF, frontal eye field.

For seed-to-seed analysis, seeds were determined by the peak *z*-values in the spatial maps of independent components of interest. Posterior cingulate cortex (−6, −54, 16) was selected as the seed for the DMN. The left inferior frontal gyrus (−52, 20, 10), left temporoparietal junction (−56, −44, 32), right inferior frontal gyrus (40, 18, −6) and right temporoparietal junction (62, −36, 38) were selected as the seeds for the VAN. The left frontal eye field (−22, −6, 62) and right frontal eye field (24, 2, 64) were selected as the seeds for the DAN. When we used two-sample *t*-test to compare brain activation in the DMN (independent component 21) between problematic smartphone users and non-problematic smartphone users, we found that the dACC (−4, −16, 32) was hyperactivated in the problematic smartphone user group. This coordinate for the dACC (−4, −16, 32) was selected as a seed. In seed-to-seed analysis, problematic smartphone users showed a different connectivity pattern from the non-problematic smartphone users. For problematic smartphone users, the dACC showed additional functional connectivity with the left temporoparietal junction. Moreover, a negative connectivity from the posterior cingulate cortex to the frontal eye field (both left and right) was only observed in problematic smartphone users but not in non-problematic smartphone users (Figure 2A and Supplementary Figure 3).

### Correlation between functional connectivity strength and psychometric measures

The functional connectivity strength between the dACC and left temporoparietal junction also correlated with the SAPS (Pearson’s *r* = 0.261, *p*-value = 0.036) (Figure 2B). There was no significant correlation between the dACC and temporoparietal junction functional connectivity strength and any psychometric measures.

## Discussion

We identified aberrant functional connectivity of the dACC with the VAN and with the DMN in problematic smartphone users. Moreover, both functional connectivities were associated with proneness to smartphone addiction. In seed-to-seed analysis, problematic smartphone users showed atypical dACC functional connectivity with ROIs of the VAN which is associated with the SAPS total scores, suggesting the importance of excessive exposure to smartphone use on brain functional connectivity. Bearing in mind that the dACC is crucial in executive control process and that the VAN is crucial in bottom-up control of attention, our findings imply that alterations in attention to external stimuli mediated by the VAN may be associated with problematic smartphone use.

One of the important functions of the dACC has been considered to be self-control and persistence, which is associated with choosing an option that is in line with one’s long-term benefit (60, 61). The importance of the dACC in self-control has been demonstrated repeatedly in its association with successful self-control in response inhibition tasks (62), intertemporal choice tasks (63) and delay tasks (64). Furthermore, the dACC is also important in cognitive control and is involved with adjustment of behavior so that one’s behavior aligns with internally maintained goals and limiting behaviors that do not align with achieving such goals (65). This is consistent with the finding that activation of the dACC produce intense feelings of will power that allows one to persevere against challenges. One theory suggest that the dACC carries value signal, which allows for decision-makers to overcome a tendency to succumb to temptations (66). In addiction studies, aberration in the dACC was associated with problems in inhibitory control for drug addicts (67, 68). Considering the role of the dACC in cognitive control, the difference in functional connectivity between resting state networks, such as the VAN and DMN with the dACC may be related to problems in self-control and difficulties in avoiding behaviors, such as using of smartphones excessively even when such behavior interferes with the users’ daily lives negatively. More importantly, the dACC is an important hub of the salience network, modulating switch between the executive control network and DMN, allowing access to attention and working memory when salient stimuli is detected (69, 70). Therefore, change in functional connectivity to salience network *via* the dACC may interfere with appropriate detection and filtering of salient stimuli, such as smartphone, when using it does not align with one’s goal at the time of use.

The VAN is activated while reorienting to an unattended object that is behaviorally relevant, but is not activated when reorientation simply occurs without behavioral relevance (71). A previous study has shown that top-down signal from anterior cingulate which forms a putative task-control network may send top-down signal to the VAN, which may be deactivated when unimportant objects appear (72). Increased functional connectivity between the dACC and VAN may suggest aberration in bottom up process through the VAN in processing reorientation to external relevant stimuli. While the VAN works as a circuit breaker to interrupt signal to the DAN, thereby changing the locus of orientation, top down signal from the dACC restricts its activation only to relevant but unattended stimuli (73). Aberration in functional connectivity between the dACC and VAN may interfere with the VAN’s selective activation to relevant stimuli, which may lead to maladaptive and excessive reorientation to a smartphone even when it is not relevant to one’s current goal or task.

Increased functional connectivity between the dACC, a crucial node in salience network, with the DMN may suggest that more resource is allocated to the DMN to facilitate internal mental processes against cognitive control. Previous findings demonstrate that the salience network typically shows a positive correlation with the executive control network and a negative correlation with the DMN, which helps allocate attention to external vs. internal stimuli (70, 74). We found that the dACC activity was increased in the DMN of problematic smartphone users compared to non-problematic smartphone users. This suggest that increased resource was allocated to the DMN, which promote internal mental process, rather than cognitive control mediated by executive control network. In line with our finding, subjects with internet gaming disorder had higher functional connectivity between the central node of salience network and areas of the DMN (75).

There are several potential limitations to our study. First, our study does not reveal whether the difference in functional connectivity in problematic smartphone users are vulnerability factors or whether they reflect an outcome of excessive smartphone use. Future studies may include longitudinal studies that involve early childhood and adolescence periods. Second, our study only includes male participants. Gender differences in addiction-like behaviors and neurobiology underlying addiction has been previously demonstrated (32, 33, 36, 37). While the results from our study may provide insight into underlying neurobiology of problematic smartphone use in male participants, it is difficult to generalize it to female participants. It would be meaningful to conduct a follow up study with female participants, which will help us identify similarities and differences between the sexes, which may explain gender differences in addictive disorders. Third, as our study included relatively small number of participants and because group ICA results are noisy, it is possible that ROIs defined from our study may not effectively overcome the limitations of ROIs defined from atlases. Follow up studies with large sample size may be helpful.

## Conclusion

In conclusion, our resting-state fMRI study found stronger functional connectivity in the dACC with the DMN and VAN of problematic smartphone users compared with non-problematic smartphone users. Furthermore, we found that the dACC showed additional functional connectivity with the VAN in problematic smartphone users. Future studies on network topology and local fMRI signal fluctuation, such as ALFF, will be necessary to further discern differences between problematic smartphone users and non-problematic smartphone users. Our findings suggest that altered attentional processing and internal mental process may play an important role in problematic smartphone use in light of the differences in functional connectivity in the VAN and DMN, which may share similar abnormalities with other addictive disorders.

## Data availability statement

The datasets presented in this article are not readily available because includes potential personal information. Requests to access the datasets should be directed to JL, humanjhl@gmail.com.

## Ethics statement

The studies involving human participants were reviewed and approved by the Institutional Review Board at Severance Hospital, Yonsei University. The patients/participants provided their written informed consent to participate in this study.

## Author contributions

MK, Y-CJ, DL, and JL: conceptualization. MK and JL: data curation. Y-CJ, DL, and JL: methodology and writing – review and editing. Y-CJ and JL: supervision. MK: writing – original draft. All authors contributed to the article and approved the submitted version.

## Footnotes

1
http://www.nitrc.org/projects/conn

2
http://www.fil.ion.ucl.ac.uk/spm

3
http://mialab.mrn.org/software/gift

## Supplementary material

Supplementary Figure 1 Independent components. **(A)** Default mode network (IC21). **(B)** Ventral Attention Network (IC37). **(C)** Dorsal Attention Network, Left (IC20). **(D)** Dorsal Attention Network, Right (IC32).

Supplementary Figure 2 Independent components. **(A)** Executive control network, left (IC28). **(B)** Executive control network, right (IC34). **(C)** Salience network (IC23).

Supplementary Figure 3 Results of seed-to-seed functional connectivity analysis. Statistical inferences were thresholded using an FDR-corrected *p*-value < 0.05. Values of each functional connectivity are presented on figure.

Supplementary Figure 4 Independent components considered to be artifacts (cerebrospinal fluid or white matter). **(A)** Independent Component 5. **(B)** Independent Component 6. **(C)** Independent Component 8. **(D)** Independent Component 10.

Supplementary Figure 5 Independent components considered to be artifacts (cerebrospinal fluid or white matter). **(A)** Independent Component 11. **(B)** Independent Component 19.
