# Supplementary figures and images for: Altered resting-state functional connectivity of the dorsal anterior cingulate cortex with intrinsic brain networks in male problematic smartphone users
Source: Front Psychiatry. 2022 Oct 3;13:1008557. doi: 10.3389/fpsyt.2022.1008557 (PMC9573940; doi:10.3389/fpsyt.2022.1008557)

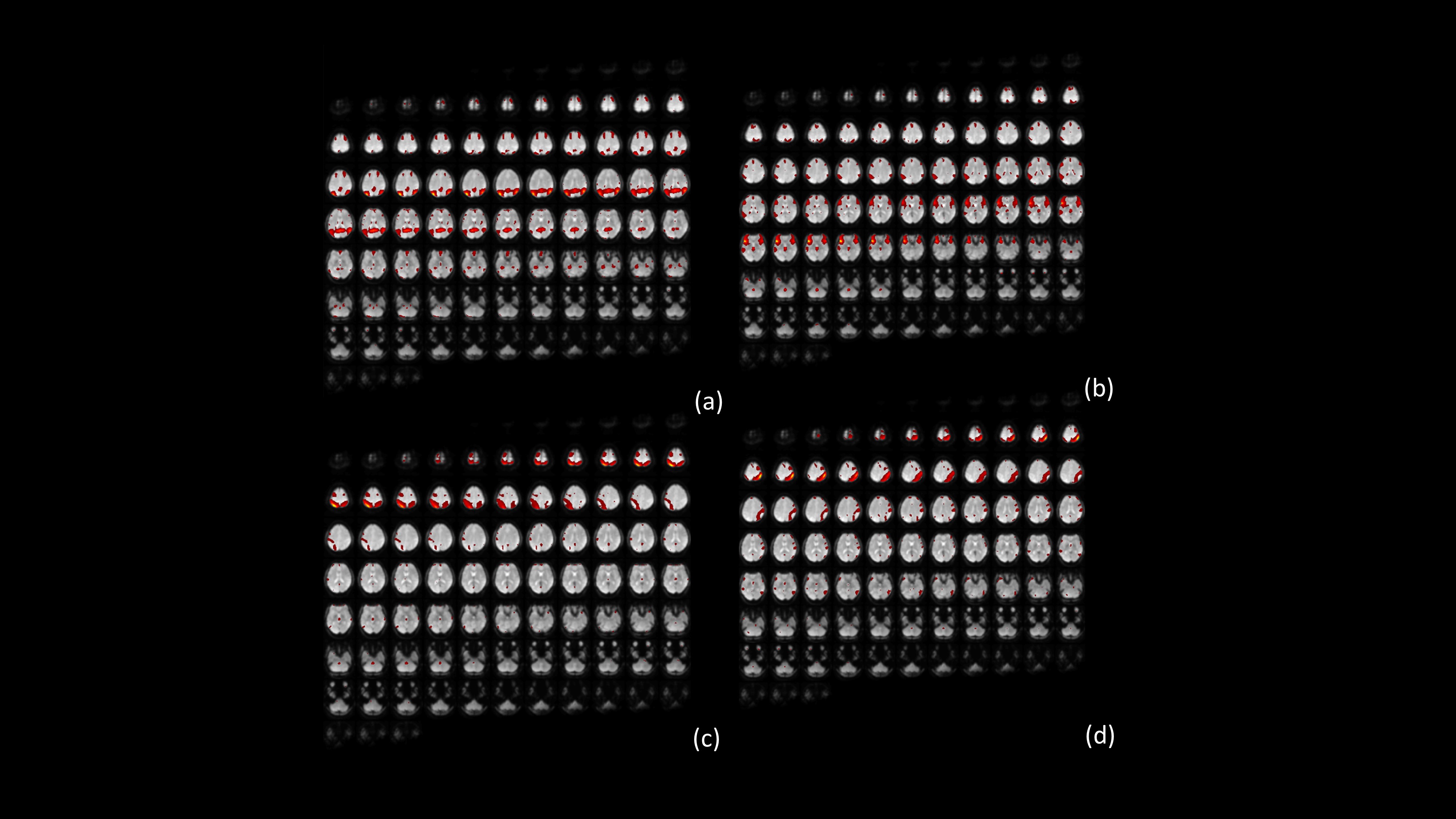

Supplement: Supplementary Figure 1 — Independent components. (A) Default mode network (IC21). (B) Ventral Attention Network (IC37). (C) Dorsal Attention Network, Left (IC20). (D) Dorsal Attention Network, Right (IC32). [file Image_1.JPEG]

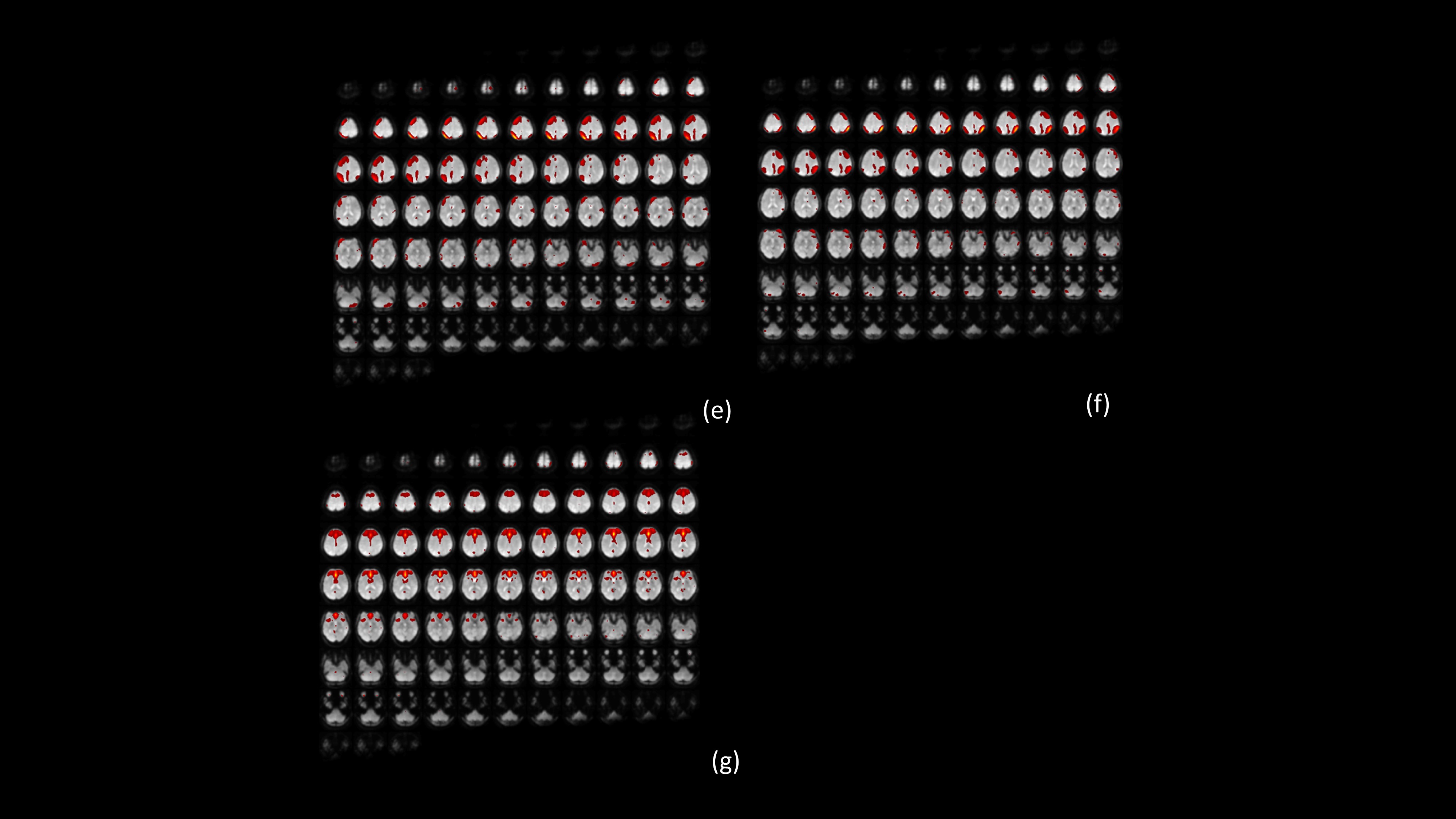

Supplement: Supplementary Figure 2 — Independent components. (A) Executive control network, left (IC28). (B) Executive control network, right (IC34). (C) Salience network (IC23). [file Image_2.JPEG]

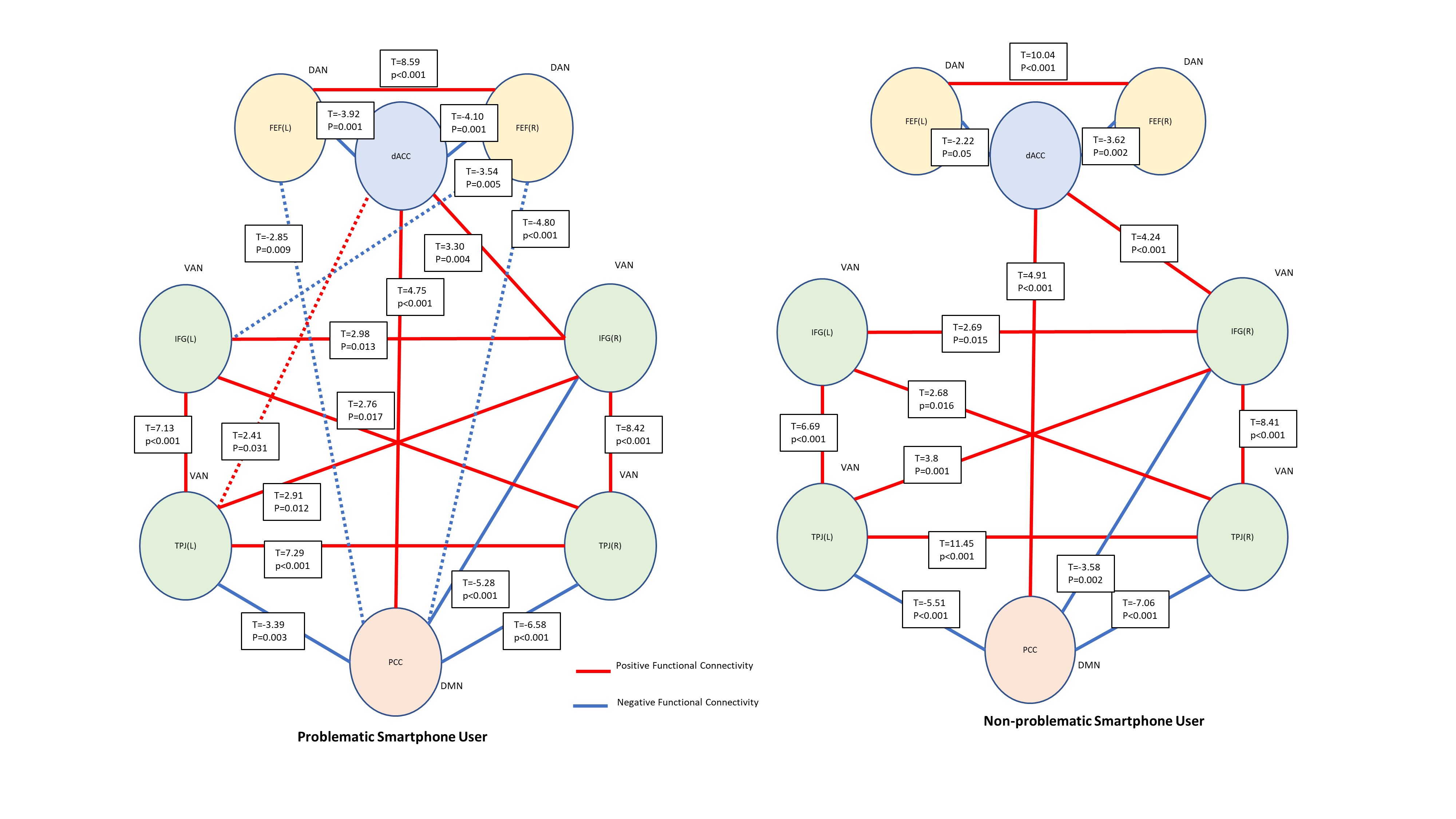

Supplement: Supplementary Figure 3 — Results of seed-to-seed functional connectivity analysis. Statistical inferences were thresholded using an FDR-corrected p-value < 0.05. Values of each functional connectivity are presented on figure. [file Image_3.JPEG]

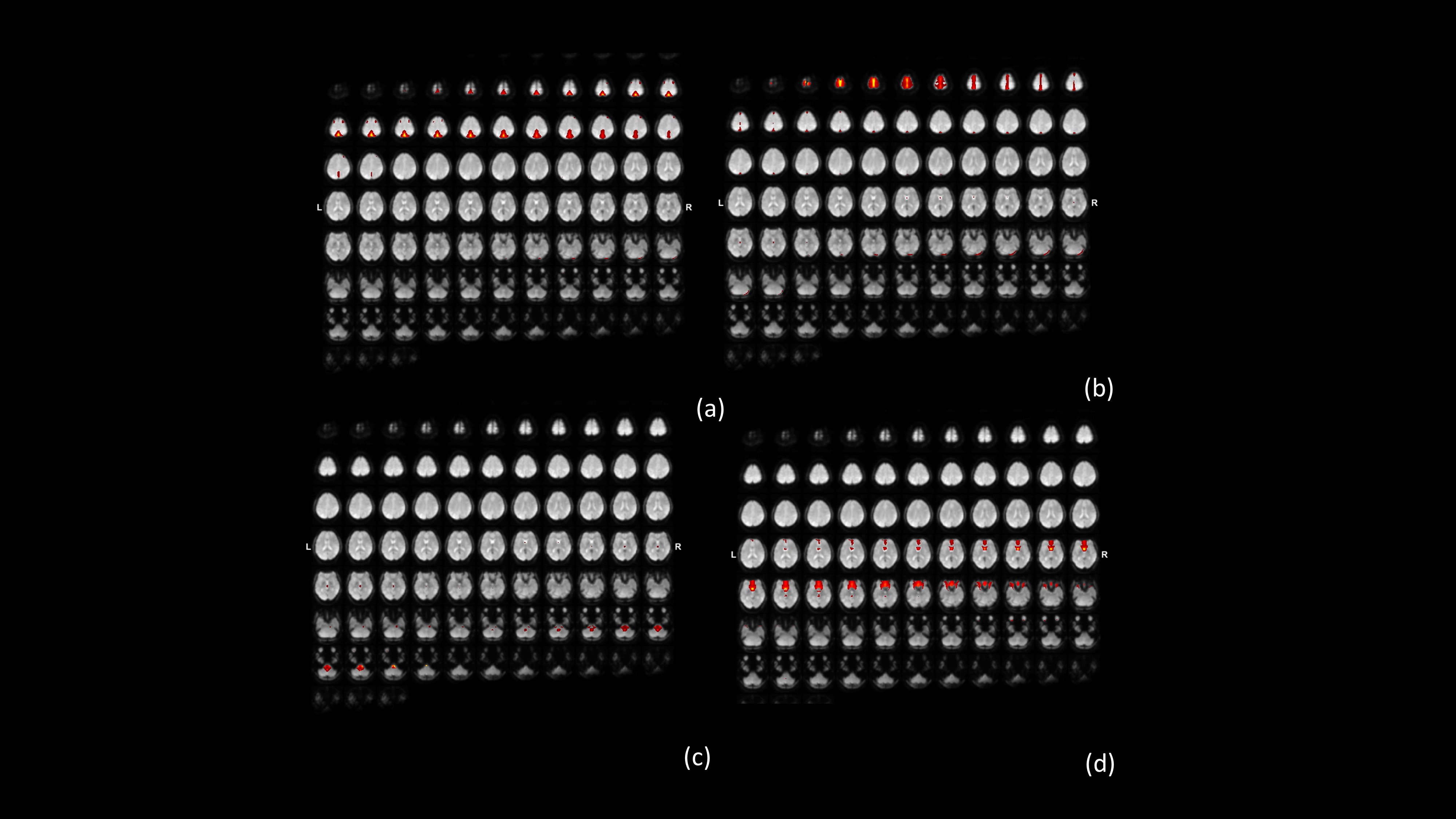

Supplement: Supplementary Figure 4 — Independent components considered to be artifacts (cerebrospinal fluid or white matter). (A) Independent Component 5. (B) Independent Component 6. (C) Independent Component 8. (D) Independent Component 10. [file Image_4.JPEG]

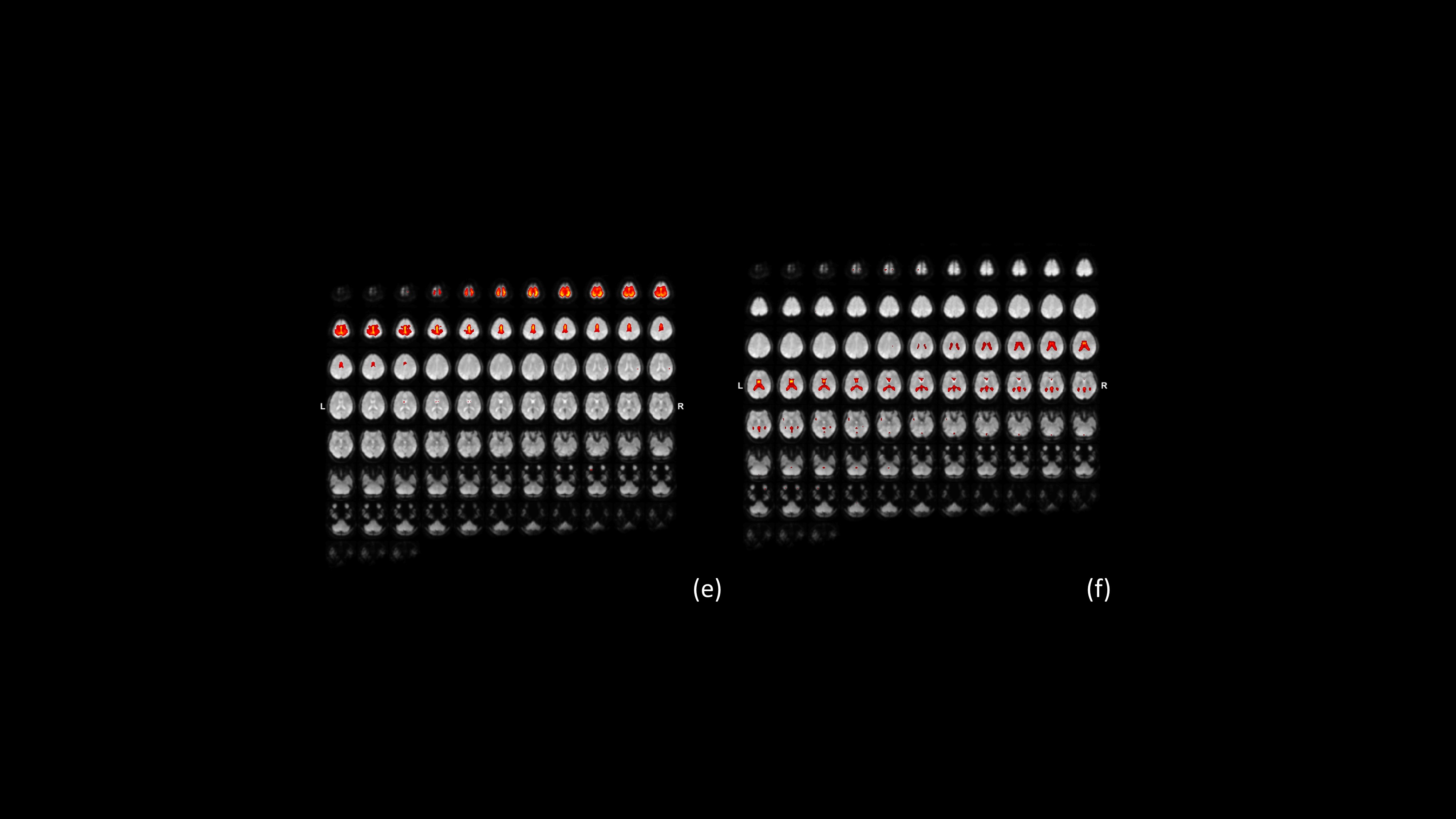

Supplement: Supplementary Figure 5 — Independent components considered to be artifacts (cerebrospinal fluid or white matter). (A) Independent Component 11. (B) Independent Component 19. [file Image_5.JPEG]
